# Genetic Syndromes Associated with Congenital Cardiac Defects and Ophthalmologic Changes - Systematization for Diagnosis in the Clinical Practice

**DOI:** 10.5935/abc.20180013

**Published:** 2018-01

**Authors:** Priscila H. A. Oliveira, Beatriz S. Souza, Eimi N. Pacheco, Michele S. Menegazzo, Ivan S. Corrêa, Paulo R. G. Zen, Rafael F. M. Rosa, Claudia C. Cesa, Lucia C. Pellanda, Manuel A. P. Vilela

**Affiliations:** Universidade Federal de Ciências da Saúde de Porto Alegre, Porto Alegre, RS - Brazil

**Keywords:** Heart Defects, Congenital/genetic, Eye Diseases, Diagnostic Tevhniques, Ophtalmologic, Heart Septal Defects, Atrial, Tetralogy of Fallot

## Abstract

**Background:**

Numerous genetic syndromes associated with heart disease and ocular manifestations have been described. However, a compilation and a summarization of these syndromes for better consultation and comparison have not been performed yet.

**Objective:**

The objective of this work is to systematize available evidence in the literature on different syndromes that may cause congenital heart diseases associated with ocular changes, focusing on the types of anatomical and functional changes.

**Method:**

A systematic search was performed on Medline electronic databases (PubMed, Embase, Cochrane, Lilacs) of articles published until January 2016. Eligibility criteria were case reports or review articles that evaluated the association of ophthalmic and cardiac abnormalities in genetic syndrome patients younger than 18 years.

**Results:**

The most frequent genetic syndromes were: Down Syndrome, Velo-cardio-facial / DiGeorge Syndrome, Charge Syndrome and Noonan Syndrome. The most associated cardiac malformations with ocular findings were interatrial communication (77.4%), interventricular communication (51.6%), patent ductus arteriosus (35.4%), pulmonary artery stenosis (25.8%) and tetralogy of Fallot (22.5%).

**Conclusion:**

Due to their clinical variability, congenital cardiac malformations may progress asymptomatically to heart defects associated with high morbidity and mortality. For this reason, the identification of extra-cardiac characteristics that may somehow contribute to the diagnosis of the disease or reveal its severity is of great relevance.

## Introduction

Congenital heart disease (CHD) is any severe structural abnormality of the heart or intrathoracic vessels that is present at birth. CHDs are considered the most common congenital malformation, significantly contributing to child mortality and morbidity, with an incidence of 4-50 cases per 1,000 births in the world.

The etiology of CHDs is still little known, and approximately 15%-20% of the cases have an unknown cause. Chromosomal abnormalities are one of the main known causes of CHDs, affecting 3-18% of the cases.^2^ Extracardiac malformations are common in patients with CHDs; defects in intra-abdominal organs and/or defects associated with genetic syndromes are observed in 7-50% of patients,^3^ increasing even more the risk of morbidity, mortality as well as of cardiac surgery. Besides, these changes may require treatment, including surgery, regardless of the cardiac problem. Among these, ophthalmological abnormalities are among the main extracardiac malformations.

Although a large number of genetic syndromes with heart disease combined with ocular manifestations have been described in the literature,^4-38^ they have not been compiled and summarized for consultation and comparison. A systematic understanding of these conditions may provide important clinical implications, contributing to the investigation and detection of abnormalities. Their diagnosis with identification of all associated conditions is crucial not only for the pediatric cardiologist seeing a patient with CHD and who should suspect ophthalmologic abnormalities, but also for the ophthalmologist who may suspect heart injury according to patients’ clinical conditions.

The aim of this study was to systematize available evidence in the literature on different syndromes that may cause CHDs associated with ocular changes, focusing on the types of anatomical and functional changes.

## Methods

A systematic review was performed on the Medline database (Pubmed, Embase, Cochrane, Lilacs). The search strategy is found in Appendix I (access the link: http://publicacoes.cardiol.br/portal/2017/abc/english/v11001/pdf/i11001014_anexo.pdf). Case reports and review studies on the association of ophthalmologic and cardiologic changes in genetic syndrome patients younger than 18 years, published until January 2016 were considered eligible. The search was performed by two independent investigators, who made a systematic analysis of titles and abstracts, and extraction of methodological characteristics, number of patients and results of all articles retrieved using the search strategy. Articles describing changes in patients older than 18 years, and articles on patients without a genetic syndrome with cardiologic and ophthalmologic changes were not considered for analysis.

This study was approved by the Research Ethics Committee of Rio Grande do Sul University Foundation of Cardiology (approval number 101593/2013-9).

## Results

**Figure 1:**
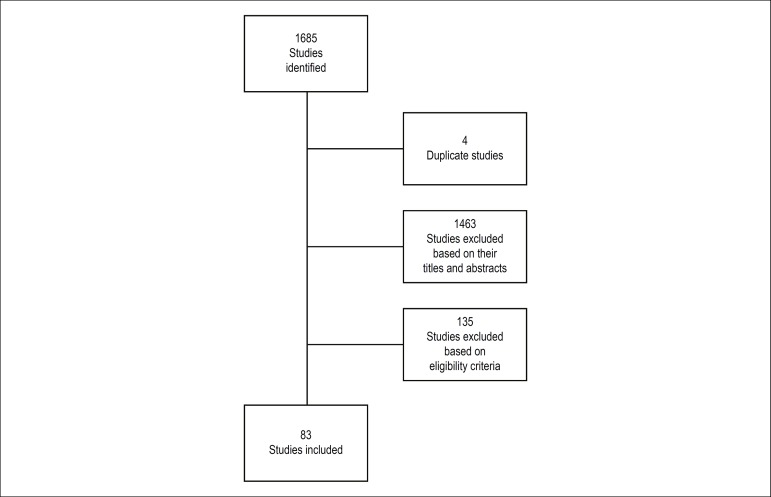
Flowchart of the studies included in this review.

A total of 1,685 articles were identified, and 83 of them, related to genetic syndromes associated with CHDs and ophthalmologic disturbances, were included in the review. Most studies were case reports (Figure 1). Tables 1 and 2 describe cardiologic changes by syndrome and ophthalmologic findings by eye segment; the most and the least common genetic syndromes can be found in Table 1 and Table 2, respectively.

**Table 1:** Common genetic syndromes associated with cardiologic and ophthalmologic disturbances

Condições Genéticas			Down	Tumer	Cat eye	Velo-cardio-facial/DiGeorge	Williams	WAGR	Rubinstein-Taybi	Alagille	Charge	Kabuki	Marfan	Noonan	Smith-Lemi-Opitz	Goldenhar	Poland-Mobius
**Cardiologic findings**		**AP**									+						
		**EP**				+	+			+				+			
		**CAo**		+													
		**EAo**					+				+			+			
		**CIA**	+	+	+	+		+	+	+	+	+					
		**CIV**	+		+		+				+	+				+	
		**DSAV**									+				+		
		**AVCI**			+												
		**DAP**												+			
		**Dext**														+	+
		**PVCSE**			+												
		**PCA**	+						+		+						
		**PVM**											+				
		**RVPA**			+												
		**TOF**	+			+			+		+						
		**VAoB**		+		+											
		**VMP**									+						
**Ophthalmologic findings**	**Extrinsic**	**An. Lacrimal**	+			+											
		**An.PabFormal/Posição**	+	+		+		+			+					+	
		**Colob. P**														+	
		**Estrabismo**	+	+	+	+	+		+		+						+
		**Hiper/Tel**	+		+						+			+		+	
		**Nistagmo**	+		+			+	+								
		**Oft. Ext**				+											+
		**PregaEp**	+	+	+									+	+	+	
	**Refractive errors and anterior segment abnormalities**	**Anir**						+									
		**Alt. Córnea e Limbo**				+				+						+	
		**Catarata ou An. de Posição**	+	+		+		+					+		+		
		**Colob. I**			+						+	+					
		**ErrosRef**	+	+	+	+								+			
		**Glaucoma**							+								
		**Hipop.I**								+							
		**M. Brush**	+														
		**Nód.I**				+											
		**ProemNC**				+											
	**Posterior segment abnormalities**	**Alt.VRet**	+	+		+											
		**An. Disco Op**								+							
		**Colob. RNC**			+						+						
		**Desc. Ret**		+									+				
		**Drus.N.Op**								+							
		**Hem.V**		+													
		**Hipop.DFO**								+							
		**Hipop.M**														+	
		**M. NeovC**									+						
		**OACRet**											+				
		**Ret.P**												+			
		**Retinob**	+														
		**DispEPR**								+							

Cardiologic findings: PA: pulmonary atresia; AIVC: absent inferior vena cava; IAC: interatrial communication; IVC: interventricular communication; CoA: coarctation of the aorta; PAD: pulmonary artery dilation; ASD: atrioventricular septal defect; AoS: aortic stenosis; PLSVC: persistent left superior vena cava; MVP: mitral valve prolapse; APVR: anomalous pulmonary venous return; TOF: tetralogy of Fallot; BAV: bicuspid aortic valve; PMV: parachute mitral valve; Ophthalmologic findings: Extrinsic: Lacrimal An.: lacrimal anomalies; Changes in eyelid shape/position; Eyelid coloboma; Hyper/Tel: hypertelorism/telecanthus; Ext.Opht: external ophthalmoplegia; EF: epicanthic fold. Refractive errors and anterior segment abnormalities: Anir: aniridia; Changes in cornea and limbus; Cataract or position abnormalities; I Colob.: Iris coloboma; RE: refractive errors; IHypop: iris hypoplasia; Brushfield spots; INod.: Iris nodules; Prom.CN: prominent corneal nerves; Posterior segment abnormalities: Abn.RV: abnormal retinal vessels; OD Abn.: optic disk abnormalities; Ret/Optic/Choroid/Nerve Colob.: Retinal/ Optic/Choroid nerve coloboma; Ret det.: retinal detachment; ODD: Optic Disk Drusen; Vit. Hem.: Vitreous hemorrhage; DH Fundus.: Diffuse hypopigmentation in the fundus; M Hypop.: macular hypoplasia; C Neov M.: choroidal neovascular membrane; Ret CAO: retinal central artery occlusion; Rret P.: retinitis pigmentosa; Retinob: retinoblastoma; spots in the retinal pigment epithelium.

**Table 2:** Rare genetic syndromes associated with cardiologic and ophthalmologic disturbances

Condições Genéticas			Adams-oliver	Alstrom	Botalli	Duane	Hutchinson-Gilford	Leber	McDonough	Mowat-Wilson	Óculo-facio-cardio-dental	Okihiro	Oto-palato-digital	Peters	PHACE	Sjogren-Larsson-like
**Cardiologic findings**		**AP**								+						
		**CIA**	+					+			+	+	+		+	
		**CIV**		+			+			+	+	+		+		+
		**CoAo**								+					+	
		**Dext**				+								+		
		**DAo**			+								+			
		**DSVa**											+			
		**DSAV**													+	
		**EVM**											+			
		**EAo**							+							
		**EVAo**								+						
		**EP**						+		+						
		**FE**						+								
		**PCA**			+					+				+	+	
		**RM**													+	
		**TOF**								+				+	+	
		**VAOB**								+						
**Ophthalmologic findings**	**Extrinsic**	**AI.CiI**					+								+	
		**An.Palp Formal/Posição**				+		+		+		+			+	
		**Estr**	+		+	+			+	+	+	+		+	+	
		**Hiper/Tel**												+		+
		**Microf**									+			+		
		**Nist**		+				+		+						
		**PregaEp**				+										
	**Refractive errors and anterior segment abnormalities**	**An.Corneana**					+							+		
		**An.Palp Forma/Posição**							+							
		**An. Pulpilares**			+			+								
		**Caratarata ou An. de Posição**									+		+	+	+	
		**Colob.I**												+		
		**ErrosRef**			+			+		+					+	+
		**Glaucoma**									+		+	+		
		**Heterl**								+					+	
		**Hipopl**													+	
	**Posterior segment abnormalities**	**Alt.VRet**					+	+						+		
		**An.Disco Óptico**						+							+	
		**Colob.C**												+		
		**DeslRet**												+		
		**Hemanl**													+	

Cardiologic findings: PA: pulmonary atresia; IAC: interatrial communication; IVC: interventricular communication; CoA: coarctation of the aorta; Dext: dextrocardia; AoD: aortic dilatation; DSVa: Dilatation of sinus of Valsalva; ASD Atrioventricular septal defect (AVSD); TMV: thickened mitral valve; AoS: aortic stenosis; AoVS: aortic valve stenosis; PS: pulmonary stenosis; EFE: endocardial fibroelastosis; PDA: patent ductus arteriosus; MR: mitral regurgitation; TOF: tetralogy of Fallot; BAV: bicuspid aortic valve Ophthalmologic findings: Extrinsic: eyelash Abn: Eyelash abnormalities; Changes in eyelid shape/position; Str: strabismus; Hyper/Tel: hypertelorism/telecanthus; Microph: microphtalmia; Nyst: nystagmus; EF: epicanthic fold Refractive errors and anterior segment abnormalities: Corneal Abn: corneal abnormality; Changes in eyelid shape/position; Pupillary Abn: pupillary abnormalities; Cataract or position abnormalities; I Colob.: Iris coloboma; RE: refractive errors; Glauc: glaucoma; IHeter: iris heterochromia; IHypop: iris hypoplasia Posterior segment abnormalities: Abn.RV: abnormal retinal vessels; OD Abn.: optic disk abnormalities; CColob: Choroid nerve coloboma; Ret det.: retinal detachment; IHeman: intraorbital hemangioma

The most frequently described genetic syndromes associated with CHD-related ocular changes were Down syndrome, velo-cardio-facial/DiGeorge syndrome, CHARGE syndrome and Noonan syndrome. The most common cardiac malformations (with different etiologies) were interatrial communication (77.4%), interventricular communication (51.6%), patent ductus arteriosus (35.4%), pulmonary artery stenosis (25.8%), and tetralogy of Fallot (22.5%). The highest number of possible cardiac repercussions was found in CHARGE (8), Cat eye (5), velo-cardio-facial (4), and Down (4) syndromes, with a mean of 2.9 cardiologic findings/syndrome.

Regarding the occurrence of concomitant ocular findings, a mean of 4.6 findings were found among the most prevalent CHDs, especially in the velo-cardio-facial, Turner, cat eye, CHARGE and Goldenhar syndrome, and of 3.5 findings among the least common diseases (Table 2), especially the Peters, Phace, Bloch and Leber syndromes. External ocular disorders are the most common manifestations, with a mean of 2.4 findings/syndrome (among the most common syndromes), particularly Down syndrome, CHARGE syndrome, cat eye syndrome and velo-cardio-facial syndrome (Table 1), and a mean of 1.38 findings among the least common syndromes, with emphasis to Bloch, Duane, Mowat-Wilson, oculofaciocardiodental, Peters and Phace syndromes (Table 2).

Refractive error was reported in Down, Turner, cat eye, velo-cardio and Noonan syndromes, as well as in eight rare syndromes (Table 2). Anterior segment of the eye was more frequently affected in the velo-cardio-facial, Down, Peters and Phace syndromes, whereas the posterior segment was more frequently affected in the Turner, Alagille, Marfan, Bloch and Peters syndromes. Ocular findings associated with these syndromes were: strabismus (43.4%), cataract (28.0%), abnormalities of eyelid position and shape (28%), nystagmus (21.7%), refractive errors (19.5%), glaucoma (19.5%), and hypertelorism (19.5%).

## Discussion

Due to their clinical variability, congenital cardiac malformations may progress asymptomatically to severe heart defects associated with high morbidity and mortality. For this reason, the identification of extra-cardiac characteristics that may somehow contribute to the diagnosis of the disease or reveal its severity is of great relevance. However, so far, few studies have investigated more specific extracardiac factors, such as ophthalmologic ones. In light of the potential associations between cardiologic and ophthalmologic changes, both cardiologist and ophthalmologist should be aware of concomitant signs that may indicate certain syndromes or their severity. Among these genetic syndromes, the most frequently described cardiac manifestations were interatrial and interventricular communications, patent ductus arteriosus, pulmonary artery stenosis, and tetralogy of Fallot, whereas the most common ocular diseases were strabismus, cataract, eyelid disturbances, nystagmus, glaucoma, refractive errors and hypertelorism. Mean number of ocular findings per genetic syndrome associated with heart disease was 3.5 among uncommon syndromes, and 4.6 among the most common syndromes.

A recent systematic review^39^ showed that few studies have assessed the prevalence of ocular findings in CHD that are not associated with genetic syndrome. The prevalence was estimated at 32.5%, with cataract, strabismus, and retinopathy as the main consequences described.^39^ In case of genetic syndromes, such estimation is limited due to the scarcity of series and reports.

Down syndrome had the highest number of patients described - more than 6,000 patients in the 6 articles analyzed. This is the most common syndrome in newborns with an incidence of 1/660 live births. In 95% of cases, Down syndrome is caused by nondisjunction during maternal meiosis I, resulting three copies of chromosome 21 in each cell; 4% of these cases are related to gene translocations and 1% to mosaicism. The frequency of CHDs in children with trisomy 21 is variable in the literature, varying from 20% to over 60%.^40^ These children are known to be prone to strabismus, hypertelorism, upslanted palpebral fissures, epicanthic fold, supernumerary retinal vessels, Brushfield spots, refractive errors, cataract, nystagmus, amblyopia.

In general, the approach of children with genetic syndrome is more complex, requiring the simultaneous involvement of many medical specialties. These children should be followed-up by a multidisciplinary staff, which would be responsible for the diagnosis, the therapeutic project and patients’ follow-up.

Among these study’s limitations, the most important is the publication bias of the reviewed articles. Although available published data do not enable a meta-analysis, the summary of these findings enables the compilation of data published in sporadic reports into a unique text, resizing the problem dimension and demanding more comprehensive studies. We performed an extensive article search, without language restrictions, and this sensitivity was a strength of this study. However, an intrinsic limitation of a systematic review is the quality of the studies included.

## Conclusion

This study demonstrated the variety of cardiologic and ophthalmologic findings associated with these genetic syndromes, emphasizing the importance of this simultaneity, and that signs in the eye and appendages and cardiac signs require an integrated approach. Since these cases can cause severe functional disturbances and high morbidity, their routine assessment should include an ophthalmologic examination. Primary detection of any of these ocular signs can determine the investigation of a so far unrecognized cardiac change.
