# Supplementary material for: Genetic Syndromes Associated with Congenital Cardiac Defects and Ophthalmologic Changes - Systematization for Diagnosis in the Clinical Practice
Source: Arq Bras Cardiol. 2018 Jan;110(1):84–90. doi: 10.5935/abc.20180013 (PMC5831306; doi:10.5935/abc.20180013)
Supplement: Supplementary file 1 [file abc-110-01-0084-suppl01.pdf]

Original Article

Appendix I - Search strategy

#1 “Heart Septal Defects, Ventricular”[Mesh] OR “Ventricular Septal Defect” OR “Ventricular Septal Defects” OR “Defect, Ventricular Septal” OR “Septal Defect, Ventricular” OR “Septal Defects, Ventricular” OR “Intraventricular Septal Defects” OR “Intraventricular Septal Defect” OR “Heart Septal Defects, Atrial”[Mesh] OR “Atrial Septal Defects” OR “Defect, Atrial Septal” OR “Septal Defect, Atrial” OR “Septal Defects, Atrial” OR “Atrial Septal Defect” OR “Persistent Ostium Primum” OR “Ostium Secundum Atrial Septal Defect” OR “Aortic Coarctation”[Mesh] OR “Aortic Coarctations” OR “Coarctation, Aortic” OR “Fontan Procedure”[Mesh] OR “Fontan Operation” OR “Hemi-Fontan Procedure” OR “Hemi Fontan Procedure” OR “Bidirectional Glenn Shunt” OR “Bidirectional Glenn Shunts” OR “Bidirectional Glenn Procedure” OR “Bidirectional Glenn Procedures” OR “Bidirectional Cavopulmonary Shunt” OR “Bidirectional Cavopulmonary Shunts” OR “Tricuspid Atresia”[Mesh] OR “Tricuspid Atresias” OR “Absent Right Atrioventricular Connection” OR “Tricuspid Valve Atresia” OR “Tricuspid Valve Atresias” OR “Right ventricle hypoplasia” OR “Right ventricular hypoplasia” OR “Isolated right ventricular hypoplasia” OR “Hypoplasia of the right ventricle” OR “Isolated hypoplasia of the right ventricle” OR “Transposition of Great Vessels”[Mesh] OR “Great Vessels Transposition” OR “Transposition of Great Arteries” OR “Great Arteries transposition” OR “Tetralogy of Fallot”[Mesh] OR “Fallot's Tetralogy” OR “Fallot Tetralogy” OR “Fallots Tetralogy” OR “heart defects, congenital”[mesh] OR “congenital heart defect” OR “heart abnormalities” OR “heart abnormality” OR “congenital heart” #2 “Visual Acuity”[Mesh] OR “Acuities, Visual” OR “Acuity, Visual” OR “Visual Acuities” OR “acuities” OR “Amaurosis Fugax”[Mesh] OR “Monocular Blindness, Transient” OR “Blindness, Transient Monocular” OR “Transient Monocular Blindness” OR “Blindness, Monocular, Transient” OR “Blindness”[Mesh] OR “Blindness, Acquired” OR “Acquired Blindness” OR “Blindness, Monocular” OR “Monocular Blindness” OR “Blindness, Hysterical” OR “Hysterical Blindness” OR “Blindness, Transient” OR “Transient Blindness” OR “Blindness, Legal” OR “Legal Blindness” OR “Amaurosis” OR “Amauroses” OR “Blindness, Complete” OR “Complete Blindness” OR "Visual Perception"[Mesh] OR "Perception, Visual" OR "Perceptions, Visual" OR “Visual Perceptions” OR “Visual function” OR “Vision, Ocular”[Mesh] OR “Ocular Vision” OR “Vision” OR “Light Signal Transduction, Visual” OR “Visual Phototransduction” OR “Phototransduction, Visual” OR “Visual Transduction” OR “Transduction, Visual” OR “Visual Light Signal Transduction” OR “Retina”[Mesh] OR “retinas” OR “Retinopathy” OR “Retinal Diseases”[Mesh] OR “Disease, Retinal” OR “Diseases, Retinal” OR “Retinal Disease” OR “Retinal vascular disease” OR "Retinal Vessels"[Mesh] OR “Retinal Vessel” OR “Vessel, Retinal” OR “Vessels, Retinal” OR “Retinal Blood Vessels” OR “Blood Vessel, Retinal” OR “Blood Vessels, Retinal” OR “Retinal Blood Vessel” OR “Vessel, Retinal Blood” OR “Vessels, Retinal Blood” OR “Pecten Oculi” OR “Vascular tortuosity” OR “Retinal vascular occlusion” OR “Stasis retinopathy” OR “Retinopathy of Prematurity”[Mesh] OR “Prematurity Retinopathies” OR “Prematurity Retinopathy” OR “Retrolental Fibroplasia” OR “Fibroplasia, Retrolental” OR “Fibroplasias, Retrolental” OR “Retrolental Fibroplasias” OR “Refractive Errors”[Mesh] OR “Error, Refractava” OR “Errors, Refractive” OR “Refractive Error” OR “Refractive Disorders” OR “Disorder, Refractive” OR “Disorders, Refractive” OR “Refractive Disorder” OR “Ametropia” OR “Ametropias” OR “Eye Injuries”[Mesh] OR “Eye Injury” OR “Injury, Eye” OR “Injuries, Eye” OR “eye lesion” OR “Amblyopia” OR “Amblyopia”[Mesh] OR “Vision, Binocular”[Mesh] OR “binocular vision” OR “Glaucoma”[Mesh] OR “Glaucomas” OR “Strabismus”[Mesh] OR “Squint” OR “Phorias” OR “Phoria” OR “Strabismus, Noncomitant” OR “Noncomitant Strabismus” OR “Mechanical Strabismus” OR “Strabismus, Mechanical” OR “Strabismus, Comitant” OR “Comitant Strabismus” OR “Convergent Comitant Strabismus” OR “Comitant Strabismus, Convergent” OR “Strabismus, Convergent Comitant” OR “Hypertropia” OR “Hypertropias” OR “Cataract”[Mesh] OR “Cataracts” OR “Lens Opacities” OR “Lens Opacity” OR “Opacities, Lens” OR “Opacity, Lens” OR “Cataract, Membranous” OR “Cataracts, Membranous” OR “Membranous Cataract” OR “Membranous Cataracts” OR “Pseudoaphakia” OR “Pseudoaphakias” OR “Optic nerve” OR “Optic nerve injuries” OR “Optic Disk” #1 AND #2
